# Advances in understanding the molecular mechanisms of borderline ovarian tumors

**DOI:** 10.3389/fmolb.2024.1429852

**Published:** 2024-08-30

**Authors:** Shiying Chen, Li Huang, Meili Liang, Yajing Xie, Zhimei Zhou, Yumin Ke, Zhuna Wu

**Affiliations:** Department of Gynecology and Obstetrics, The Second Affiliated Hospital of Fujian Medical University, Quanzhou, Fujian, China

**Keywords:** bot, clinical detection methods, diagnosis, molecular mechanism, treatment

## Abstract

Borderline ovarian tumors (BOTs), which are a special type of epithelial tumor of the ovary, lie between benign and malignant tumors and have low malignant potential. Due to the fact that the early symptoms of these tumors are relatively subtle, they are not easy to diagnose clinically. This study explores advancements in clinical detection methods and provides a comprehensive overview of molecules such as cell migration factors, cell transcription factors, cell damage repair factors, cell cycle regulators, and tumor suppressor genes that are related to the development of BOTs and their related mechanisms in recent years, thus aiming to provide more sensitive, specific, and efficient differential diagnosis and treatment plans for patients to improve their prognosis and survival outcomes.

## 1 Introduction

BOTs, accounting for 10%–20% of epithelial ovarian tumors, which represent a prevalent gynecological condition, exhibit slow growth; however, they possess the potential to metastasize to distant sites and eventually progress into invasive cancer. BOTs include six histological subtypes (serous, mucinous, seromucous, endometrioid, clear cell, Brenner’s), of which serous and mucinous are the most frequent (Bourdel et al., 2021). Due to the absence of evident clinical symptoms or signs during the early stage and the lack of specific screening methods, most patients are typically identified through physical examinations or when they present with abdominal pain and abdominal distension, among other symptoms. Therefore, the early detection and treatment of this disease remain the current research focus.

BOTs represent a distinct pathological type that falls between benign ovarian tumors and ovarian cancer. Their management differs from that of both entities. The median age of onset is 46 years, and the 5-year and 10-year survival rates are 95% and 90%, respectively. Although the overall survival rate is high, the outcomes remain unsatisfactory for a subset of patients characterized by high recurrence rates and implantation metastasis.

Recent research on the molecular basis of tumorigenesis and progression has become increasingly mature. Mutations in proto-oncogenes and tumor suppressor genes, epigenetic changes such as DNA methylation and histone modification, abnormal gene expression regulation at the transcription or translation level or miRNA abnormal regulation, and abnormal signal transduction pathways, among other factors, all play a role in some way in the formation of tumors. Given the difficulty of diagnosing BOTs, we attempt to summarize and elucidate the relevant mechanisms of their development and progression from these aspects, with the hope of gaining a deeper understanding of BOTs.

## 2 Auxiliary examination

Currently, the preoperative clinical diagnosis of BOT surgery often involves the use of laboratory tests, such as tumor markers, and comprehensive imaging modalities, including ultrasound, CT, and MRI. The ultrasound technique remains the most commonly employed early screening and adjunctive method; however, its diagnostic accuracy for BOTs is limited to 60%–70%. Tumor infiltration may be associated with the number of lesions, resistance index values, and blood flow signals detected on ultrasound, thereby offering some guidance for tumor prognosis to a certain extent. CT imaging can effectively depict the solid components, extent, and morphological characteristics of ovarian tumors, whereas MRI offers superior resolution for visualizing soft tissues, multiple quantitative parameters, and multisequence imaging capabilities. Both techniques are widely employed for assessing tumor morphology and aiding in tissue classification; however, their diagnostic utility remains somewhat constrained (Verdecchia et al., 2021; Borrelli et al., 2017).

Recent studies have identified an increasing number of additional potential markers for tumor diagnosis and differentiation. Despite its widespread use as a preoperative biomarker for ovarian tumors, CA125 levels exhibit limited sensitivity and specificity. The ROMA index is a composite measure derived from CA125, HE-4, and menopausal status. In contrast, the CPH-I index (CPH-I = −14.0647 + 1.0649 × log2(HE-4) + 0.6050 × log2(CA125) + 0.2672 × age/10, PP (%) = e (CPH-1)/[1 + e (CPH-1)], where PP > 7% indicates high risk) represents a novel tumor index that incorporates age as a variable instead of menopausal status. Multiple studies have consistently demonstrated the superior diagnostic value of the CPH-I over CA125 in differentiating BOTs and early-stage ovarian cancer (Wang et al., 2019). Ke Huang and colleagues proposed a methodology that integrates inflammation biomarkers and tumor biomarkers, whereby they posited that the ratios of neutrophils to lymphocytes (NLR) and platelets to lymphocytes (PLR) are associated with tumor progression and prognosis. They further determined the optimal cutoff values for each indicator corresponding to discrimination. The diagnostic system combining CA125 with the NLR and PLR was assessed through the construction of a multivariate logistic regression model in this study. The results demonstrated that this diagnostic system exhibited superior accuracy in distinguishing the nature of epithelial ovarian tumors (EOTs) compared to single or dual combinations, particularly for borderline epithelial ovarian tumors (BEOTs) (Huang et al., 2023). A comparative study was conducted to investigate the serum free amino acid (SFAA) profiles of different types of ovarian tumors. The findings demonstrated a significantly lower expression level of histidine in ovarian borderline and malignant tumors than in benign ovarian tumors, thus suggesting its potential diagnostic value (AUC = 0.787) (Horala et al., 2021).

The proliferative activity of tumors is widely employed to assess the cell growth fraction and prognosis of patients. Ki-67, which is a representative nuclear antigen associated with cell proliferation, plays a crucial role throughout the process of mitosis and is expressed in nearly all cells (except during the G0 phase). This characteristic has significant implications for determining tumor aggressiveness and prognostication. Klaudiusz Ciepliński et al. employed immunohistochemical techniques to assess the expression levels of several proliferation markers in 42 cases of borderline tumors derived from the pathological tissues of patients. The findings of this study revealed distinct expression rates for Ki-67 (n = 25/42), MCM3 (n = 38/42), PCNA (n = 28/42), and topoisomerase IIα (14/48), with a negative correlation observed between Ki-67 immunoreactivity and patient age. Additionally, the study demonstrated significant upregulation of Ki-67 and topoisomerase IIα expression in tumor tissues with a diameter exceeding 10 cm. Moreover, a positive correlation was found between Ki-67 and both topoisomerase IIα and MCM3. These findings suggest that simultaneous detection using multiple markers can increase the precision of BOT diagnosis (Ciepliński et al., 2018).

According to the statistical data, the recurrence rate of BOTs ranges from approximately 5%–20%. Numerous studies have been conducted to investigate and validate the clinical characteristics and prognostic factors associated with BOT recurrence. L.M. Stewart. et al. reported of a significant correlation between pelvic inflammatory diseases, infertility, ectopic pregnancy, and serous ovarian borderline tumors (sBOTs), thus providing support for the hypothesis that an underlying inflammatory process may be involved in the etiology of sBOTs (Stewart et al., 2020). Sozen et al. (2018) identified an age threshold younger than 35 years, the presence of microvillous structures, implantation phenomena, and fertility-preserving surgery as being predictive factors for the recurrence and progression of BOTs. Besides, advanced FIGO staging at a later stage, along with elevated CA125 levels, are also considered autonomous risk factors for the recurrence of BOTs (Niu et al., 2021). Although the prognosis of most BOT patients is favorable, individuals with the aforementioned high-risk factors must be closely monitored so that any recurrences can be detected and promptly treated.

## 3 Etiology, related molecules and mechanisms of BOTs

**FIGURE 1:**
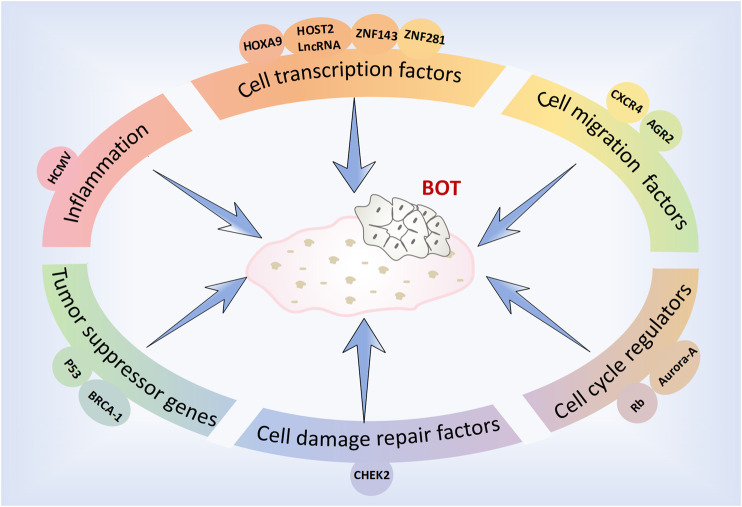
Factors and molecules associated with the development of BOT.

The factors, molecules and related pathways involved in the development and progression of BOTs are complex and diverse (Figure 1). To effectively and specifically differentiate and accurately diagnose these patients, we have summarized recent studies pertaining to the diagnosis and treatment of BOTs.

### 3.1 Inflammation and BOTs

Previous studies have demonstrated a significant association between chronic inflammation and the progression of tumors. Women diagnosed with pelvic inflammatory disease (PID) exhibit increased susceptibility to developing BOTs, and human cytomegalovirus (HCMV) has been identified as being a potential etiological agent in certain cases. HCMV infection can induce the upregulation of 5-lipoxygenase (5LO), which is a potent inflammatory mediator, thereby promoting inflammation, stimulating cellular proliferation, and activating antiapoptotic signaling pathways. Tests were conducted on a series of BOT tissue and blood samples to detect and analyze HCMV proteins. They observed widespread expression of HCMV protein, IE in 87% of BOT tissues, expression of HCMV pp65 in 40% of tissues, expression of 5LO in 90% of tissues, and significantly elevated levels of HCMV IgG antibodies in blood samples from BOT patients. Their initial hypothesis suggested that HCMV may induce inflammation and contribute to the pathogenesis of BOTs. Further research and validation are required to investigate whether antiviral therapy can effectively improve the prognosis of BOT patients (Rahbar et al., 2020).

### 3.2 Cellular molecules and the BOT

With the advancement of molecular biomedicine, research on molecular biology pertaining to BOTs has reached a state of increasing maturity, thus aiding in elucidating diverse cellular molecules and signaling pathways implicated in the development of BOTs. We classified BOTs related cell molecules into cell migration factors, cell transcription factors, cell damage repair factors, cell cycle regulators, and tumor suppressor genes. Each molecule’s expression level in different types of ovarian tumors as well as possible related pathogenic mechanisms and signaling pathways were summarized (Table 1).

**TABLE 1:** Summary table of the expression of different molecules in various types of ovarian tumors and possible related pathogenesis and signaling pathways.

Molecules		Expression levels in different type of ovarian tumors				Pathogenic mechanisms and signal pathway that molecules may participate in
		Normal ovarian epithelium	Benign ovarian tumors	BOTs	Malignant ovarian tumors	
CXCR4		0% Rahbar et al. (2020)	15% Rahbar et al. (2020); Guo et al. (2011)	40%–50% Rahbar et al. (2020); Guo et al. (2011)	53.8%–80% ^[^ Rahbar et al. (2020); Guo et al. (2011)	• PI3K- AKT↑ • MAPK↑ • JAK/STAT?
AGR2		0%–31% Moidu et al. (2020)	66.7%–76% in serous Moidu et al. (2020); Armes et al. (2013) 100% in mucinous Moidu et al. (2020)	95%–100% in serous Moidu et al. (2020); Armes et al. (2013) 100% in mucinous Moidu et al. (2020)	70% in serous Armes et al. (2013) 100% in mucinous Moidu et al. (2020)	• EGFR, VEGF, FGF2↑ • ERK1/2-MAPK↑ • DNA methylation of the AGR2 promoter↑ • p53 pathway↓
HOXA9		Methylated HOXA9 expression level Guo et al. (2017):				• PI3K- AKT↑ • TGF β2-MSCs/CAFs↑ • NF-κB↓ • HIF-1↓ • JAK/STAT?
		—	0.35%	9.6%	35%	
HOST2 lncRNA		F value (Target gene copy number/reference gene copy number)Tang et al. (2022)				• Let-7b↓/CDK6,RB1↑ • JAK/STAT3↑
		0.12 ± 0.02	—	0.99 ± 0.09	1.05 ± 0.10	
ZNF family	ZNF143	—	—	90% in sBOTs and LGOC Hahn and Hermeking (2014)		• MDIG/CDC6↑ • ZNF143-FBXO9↑- FBXW7↓
	ZNF281	—	—	57% in sBOTs and LGOC Hahn and Hermeking (2014)		• ZNF281- NANOG/SOX2/OCT4- LGR5/CD133↑ • ZNF281-CDH1/ OCLN/CLDN7-EMT↑
CHEK2		A missense mutation (c.470T >C) probability is approximately two-fold in BOTs compared with normal control Kabacik et al. (2011)				• ATM-CHEK2-P53↑
Rb	Rb/p105	Nuclear expression Amin et al. (2015)				• P16-CDKs-Rb-E2F↑
		—	—	50% in mBOTs 84.6% in sBOTs	—	
	Rb2/p130	Nuclear expression Amin et al. (2015)				
		—	—	10% in mBOTs 80.8% in sBOTs	—	
		Cytoplasmic expression Amin et al. (2015)				
		—	—	80% in mBOTs 0% in sBOTs	—	
		Subcellular distribution patterns Amin et al. (2015)				
		—	nuclear expression	nuclear-cytoplasmic expression	cytoplasmic expression	
Aurora A kinase		Expression level and subcellular distribution patterns He et al. (2008)				• E2F3- Aurora A kinase-NFκB-IL- 6/TGFα/MCP1↑
		—	93.5% exhibit moderate-to-heavy nuclear expression	48% exhibit nuclear expression and were intermediate between the two groups	47% exhibit weak-to-moderate perinuclear cytoplasmic staining	
P53		Immunoreactivity staining scores (*p* < 0.05 in each two group) Chefetz et al. (2014):				• P14-HdM2-P53↓- OPG/GADD45/Fax/Bax/ PUMA/P21CDKN1A↓
		—	Serous:0.93 ± 0.33	sBOTs: 2.89 ± 0.49	Serous: 4.71 ± 0.54	
BRCA-1		The incidence of decreased nuclear staining intensity in different ovarian tumor types compared with normal tissue (all have positive expression)				• BRCA1-P53 pathway↑ • BRCA1-CHEK1↑ • BRCA1/BRCA2/Rad51- DSBS↑ • BRCA1- NBS1/Rad50/Mre11↓- DSBS↑ • BRCA1-CAbl-P73/Rad↑
		—	35.82% (of which serous was 34%)	39.3% (of which sBOTs was 46%)	65.4% (of which serous was 74%)	

Annotation: 1) sBOTs: serous borderline ovarian tumors. 2) mBOTs: mucinous boederline ovarian tumors. 3) ↑indicates promote or activate. 4) ↓indicates inhibit or inactivate.

#### 3.2.1 Cell migration factors

##### 3.2.1.1 CXCR4

**FIGURE 2:**
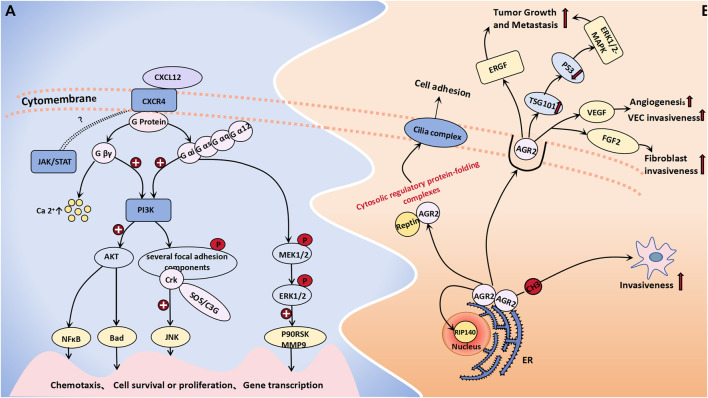
Cell migration molecules associated with BOT development. **(A)** When CXCL12 binds to CRCX4, the latter selectively engages G protein-coupled 7 transmembrane receptors on target cells, leading to its dissociation into Gβγ and Gα subunits. The former exerts a profound influence on intracellular calcium concentration elevation. Both Gβγ and Gα subunits collaboratively activate the PI3K-AKT pathway and phosphorylate diverse focal adhesion molecules. Sequentially, Gα proteins can concurrently trigger MEK1/2 activation followed by ERK1/2 activation. The JAK/STAT signaling cascade is believed to be a CXCR4-related G protein-independent signaling pathway. **(B)** AGR-2 interacts with nuclear DNA binding proteins RIP140 and Reptin, forming a cytosolic regulatory protein folding complex with the latter to facilitate cilia assembly on the cell membrane for the regulation of cell adhesion and growth. The extracellular activities of AGR2 impact the EGFR growth stimulation pathway, promote the generation of exosomes, as well as ubiquitin ligase family member TSG101, which inhibits p53 function thereby activating the ERK1/2-MAPK pathway to promote tumor growth and metastasis. DNA methylation of its promoter promotes an aggressive phenotype in tumor cells. Additionally, AGR2 enhances VEGF and FGF2 activity through homodimerization to promote angiogenesis and invasiveness.

CXCR4 is a chemokine receptor that selectively binds to G protein-coupled seven transmembrane receptors on target cells, thereby inducing chemotaxis of NK cells, T lymphocytes, and other immune cells (Guo et al., 2011). Additionally, it plays a crucial role in regulating various essential cell signaling pathways, such as the EGFR, PI3K, MAPK, and NFκB pathways, thus ultimately facilitating cellular migration. CXCR4 expression is significantly upregulated in ovarian tumor cells, with a notably greater level observed in ovarian cancer and borderline tumors than in benign tumors. One study observed that CXCR4 was highly or moderately stained in the cytoplasm of 50% of borderline tumors and 53.8% of malignant tumors, although there was no significant difference in the expression rate of the two (Pils et al., 2007). Another study showed that the positive rate of CXCR4 in malignant epithelial tumors was significantly higher than that in normal ovarian epithelial tumors, benign epithelial tumors and borderline epithelial tumors (CXCR4:80% vs. 0, 15%, 40%) (Guo et al., 2011). However, it is worth confirming that CXCR4 may be used as a sensitive factor to distinguish between benign and borderline ovarian tumors. KM Archibald conducted an expression profile analysis of HGSOC (high grade serous ovarian cancer) and demonstrated that the amplification of the *CXCR4* gene locus may represent an early event in the pathogenesis of HGSOC, which is closely associated with chromosomal instability (Archibald et al., 2012). Current research on the molecular mechanisms underlying *CXCR4* in tumorigenesis and progression has reached a state of increasing maturity. LIN HE et al. employed miR-9 shRNA transfection as a therapeutic approach for ovarian cancer cells, which resulted in a significant reduction in the expression level of CXCR4 mRNA within the transfected group. Moreover, this intervention led to a notable decrease in cell proliferation capacity. Consequently, their findings suggest that miR-9 may exert inhibitory effects on ERK1/2 and MMP-9 expression by targeting the SDF-1/CXCR4 pathway, thereby impeding the development and progression of ovarian tumors (He et al., 2017). The CXCL12/CXCR4 pathway is believed to not only modulate the biological behaviors of tumor cells but also orchestrate the metastatic dissemination of CXCR4-positive tumor cells toward organs or tissues that express CXCL12. This pathway plays a pivotal role in recruiting monocytic myeloid-derived suppressor cells (mMDSCs) to the tumor microenvironment of ovarian cancer patients, thereby impeding the efficacy of immune responses through recruitment of these immunosuppressive cells (Obermajer et al., 2011). When CXCL12 binds to CRCX4, the latter can associate with the inner surface of the cell membrane G protein, thus leading to its dissociation into the Gβγ and Gα subunits. The former type exerts a significant impact on intracellular calcium concentration elevation. Simultaneously, both the Gβγ and Gα subunits collaboratively activate PI3K, which subsequently phosphorylates various focal adhesion molecules (such as Pyk-2, P130cas, FAK, Crk-L, Nck, and Crk) and triggers the activation of AKT along with its downstream signaling molecules (Teicher and Fricker, 2010). Among these molecules, Crk can bind to SOS or C3G through its SH3 structure, thereby activating the c-Jun N-terminal kinase (JNK), which plays an important role in signal transduction. Additionally, Gα proteins can activate MEK1/2 and ERK1/2 in a sequential manner, thereby initiating the phosphorylation of diverse cellular proteins and transcription factors, including P90RSK and MMP9. This activation cascade synergistically promotes cell proliferation, invasion, and angiogenesis in conjunction with the PI3K-AKT pathway (Teicher and Fricker, 2010). CXCR4 is also postulated to participate in a G protein-independent signaling pathway, which potentially involves the JAK/STAT signaling cascade; however, further investigation is warranted to elucidate the precise underlying mechanism that is involved (Figure 2A).

##### 3.2.1.2 AGR2

AGR-2, which functions as an endoplasmic reticulum protein disulfide isomerase, can enhance cell migration and induce epithelial–mesenchymal transition. Moreover, it is significantly upregulated in various epithelial cancers, thereby contributing to the development and progression of diverse malignant tumors (Moidu et al., 2020). Research by Armes, Jane E. et al. indicated that AGR2 is expressed in all mucinous ovarian tumors (including benign borderline and malignant tumors), in approximately 95% of serous BOTs and 76% of serous cystadenomas, but only 31% in normal ovarian epithelium (Armes et al., 2013). Meanwhile, the protein level of AGR2 in the blood of ovarian cancer patients, including early-stage ovarian cancer patients, are significantly elevated compared to those in the blood of the general population (Edgell et al., 2010). Immunohistochemical staining of AGR2 was also performed on different ovarian tissues, showing that AGR2 was expressed in 78.6% of ovarian carcinomas, 100% of BOTs and 0% of normal ovarian epithelium. The overexpression of AGR2 can facilitate cell proliferation and migration (Edgell et al., 2010). AGR2 can exist as either a monomer or a dimer within the endoplasmic reticulum. It not only exhibits binding affinity toward membrane receptors but also has the ability to interact with nuclear DNA binding proteins such as RIP140 and Reptin (Brychtova et al., 2015). Furthermore, AGR2 can form a cytoplasmic regulatory protein folding complex in conjunction with Reptin, thereby facilitating the assembly of cilia complexes on the plasma membrane and ultimately regulating cell adhesion and growth (Brychtova et al., 2015). The extracellular functional activity of AGR2 has been demonstrated to impact the EGFR growth stimulation pathway and promote the generation of exosomes, as well as the ubiquitin ligase family member TSG101, which inhibits p53 function and activates the ERK1/2-MAPK pathway to facilitate tumor growth and metastasis. The utilization of monoclonal antibodies targeting AGR2 can effectively enhance tumor cell apoptosis (Brychtova et al., 2015; Negi et al., 2019). A murine model was established to investigate the metastasis of human ovarian cancer, thus demonstrating an increase in mRNA expression and a decrease in methylation at the CpG site within the *AGR2* gene promoter region in metastatic tumor tissue. These findings suggest that DNA methylation of the *AGR2* promoter plays a regulatory role in promoting a more aggressive phenotype in tumor cells (Sung et al., 2014). H Guo et al. demonstrated that AGR2 can also facilitate angiogenesis and the invasion of endothelial cells and fibroblasts by augmenting the activity of vascular endothelial growth factor (VEGF) and fibroblast growth factor 2 (FGF2). This enhancing effect is contingent upon the homodimerization domain of AGR2; thus, monoclonal antibodies targeting AGR2 self-dimerization may emerge as novel therapeutic agents for tumor treatment (Guo et al., 2017) (Figure 2B).

#### 3.2.2 Cell transcription factors

##### 3.2.2.1 HOXA9

**FIGURE 3:**
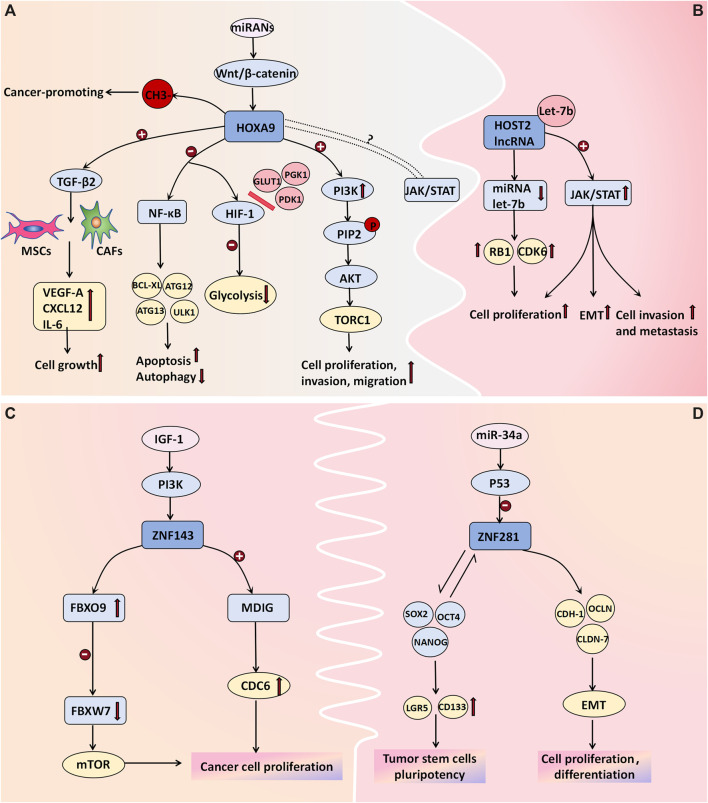
Cell transcription molecules associated with BOT development. **(A)** The methylation of HOXA9 DNA is proposed as a molecular mechanism in ovarian tumor development. Transcriptional activation of HOXA9 promotes the expression of TGF-β2, which then acts on MSCs and CAFs to induce the expression of VEGF-A, CXCL12, and IL-6 for cell growth facilitation. Deregulation in HOXA9 expression not only promotes cell apoptosis and inhibits autophagy by affecting NF-κB pathway-related genes like BCL-XL, ATG12, and ATG13 but also hinders glycolysis by blocking the binding between hypoxia-inducible factor (HIF-1) and glucose metabolism-related genes such as GLUT1, PGK1, and PDK1. Overexpression of HOXA9 upregulates molecules associated with the PI3K/AKT/mTOR pathway to promote cell proliferation and invasion. Additionally, some miRNAs target HOXA9 while its deregulation often correlates with alterations in the Wnt/β-catenin pathway. Furthermore, there is believed to be a synergistic effect between HOXA9 and the JAK/STAT pathway; however, further investigation is needed to determine the specific mechanism. **(B)** HOST2 lncRNA has a binding site for the tumor suppressor Let-7b. Upon binding, Let-7b sequesters itself and leads to increased expression of its target genes, such as CDK6 and RB1. Moreover, lncRNA HOST2 may facilitate tumor cell epithelial-mesenchymal transition (EMT), proliferation, invasion, and metastasis by activating the JAK2/STAT3 signaling pathway. **(C)** ZNF143 directly activates MDIG transcription, promoting cancer cell proliferation and colony formation by enhancing CDC6 expression. The ZNF143-FBXO9-FBXW7 signaling axis may be involved in tumorigenesis pathways. The expression of ZNF143 in ovarian tumors is regulated by the IGF-1-PI3K signaling pathway; however, further investigation is needed to understand the underlying molecular mechanisms. **(D)** ZNF281 regulates the expression of CDH-1, OCLN, and CLDN-7, along with other effector genes, to actively participate in epithelial-mesenchymal transition (EMT), cellular proliferation, and differentiation. Additionally, it interacts with transcription factors NANOG, SOX2, and OCT4 to induce stem cell markers LGR5 and CD133 expression for the formation and maintenance of tumor stem cells’ pluripotency. P53-mediated miRNAs negatively regulate ZNF281.

The HOX gene family, which consists of 39 genes arranged in four clusters (*HOXA, HOXB, HOXC, and HOXD*), is widely recognized as being a pivotal regulator of tumor development. Specifically, the *HOXA* gene cluster coordinates the development and formation of Muller’s system during embryonic development. The transcription factor *HOXA9* regulates diverse cellular activities by facilitating cell signal transduction. *HOXA9*-methylated DNA has been proposed to be a biomarker associated with ovarian tumors. The average percentage of methylated *HOXA9* in normal ovarian tissue ranges from 0% to 0.07% (average: 0.02%), as reported by Faaborg et al. (2021). Their study demonstrated significantly elevated expression levels of *HOXA9*-methylated DNA in both malignant and borderline ovarian tumors. In malignant tumors, the average proportion of methylated *HOXA9* is 35%, whereas it is 9.6% in BOTs and merely 0.35% in benign tumors, thus highlighting its potential utility as a specific biomarker for distinguishing benign ovarian neoplasms (Faaborg et al., 2021). Ko et al. (2014) proposed that the upregulation of HOXA9 in high-grade serous ovarian cancer can induce the differentiation of macrophages into tumor-associated macrophages (TAMs), which is characterized by the M2 phenotype and which is known to suppress antitumor immune responses; moreover, it is often associated with unfavorable prognosis. The pro-proliferative effect of *HOXA9* is primarily mediated through its transcriptional activation of *TGF-β2* gene expression, thus leading to the induction of *VEGF-A*, *CXCL12*, and *IL-6* in fibroblasts and mesenchymal stem cells (Ko et al., 2012). Deregulation of HOXA9 expression not only induces cell apoptosis and inhibits autophagy by modulating NF-κB pathway-related genes (including *BCL-XL*, *ATG12*, and *ATG13*) but also suppresses glycolysis by impeding the interaction between hypoxia-inducible factor (*HIF-1*) and glucose metabolism-associated genes such as *GLUT1*, *PGK1*, and *PDK1*. Moreover, its overexpression upregulates molecules within the PI3K/AKT/mTOR pathway to facilitate cell proliferation and invasion. *HOXA9* is targeted by several miRNAs, and its deregulation frequently correlates with alterations in the Wnt/β-catenin pathway. Moreover, HOXA9 is believed to exhibit synergistic effects on the JAK/STAT pathway; however, the precise underlying mechanism requires further investigation (Tang et al., 2022). Targeting the expression of HOXA9 may be one of the means of cancer treatment. However, due to the limited research data recently, the signal pathway and mechanism of *HOXA9* in the occurrence and development of BOTs still need to be further explored (Figure 3A).

##### 3.2.2.2 HOST2 lncRNA

The expression of HOST2 lncRNA is significantly upregulated in ovarian borderline cells and cancer cells, thus surpassing the levels observed in normal or benign ovarian tissues (Jing et al., 2015). Moreover, it plays a pivotal role in promoting proliferation and conferring cisplatin resistance to ovarian cancer cells. Xu et al. (2019) discovered that SH, which is a derivative of Sinomenium acutum, exhibits potential antitumor effects by suppressing the expression of HOST2 in ovarian tumor cells. These findings suggest a close correlation between the expression level of HOST2 in ovarian cancer tissues and individual patient prognosis, thus highlighting its potential as being a molecular marker for the early diagnosis of borderline ovarian tumors and ovarian cancer. Furthermore, A binding site was identified for the tumor suppressor Let-7b on HOST2. Upon binding, Let-7b sequesters itself and leads to increased expression of its target genes, such as CDK6 and RB1. This upregulation reduces translation inhibition and degradation of the target mRNA, thus ultimately altering the biological behavior of tumor cells. These findings have been experimentally validated in ovarian cancer cells and breast cancer cells (Zhang et al., 2018; Gao et al., 2014). Yang Wu et al. demonstrated the downregulation of HOST2 expression in hepatocellular carcinoma cells, thus resulting in a significant reduction in the levels of JAK2, STAT3, and vimentin while upregulating E-cadherin expression. Consequently, cell proliferation and invasion were markedly attenuated, thus suggesting that HOST2 may facilitate tumor cell epithelial–mesenchymal transition (EMT), proliferation, invasion, and metastasis through modulation of the JAK2/STAT3 pathway (Wu et al., 2018). However, the validation of this mechanism in ovarian tumor cells remains incomplete (Figure 3B).

##### 3.2.2.3 EMT-related transcription factors *ZNF281* and *ZNF143*

In recent years, emerging research has demonstrated the pivotal role of EMT in the initiation, progression, metastasis, and chemoresistance of tumors. EMT facilitates the loss of epithelial cell polarity and characteristic features such as basement membrane adhesion while conferring enhanced migratory and invasive properties, antiapoptotic potential, and extracellular matrix degradation capabilities associated with a mesenchymal phenotype. *ZNF281*, which is an epithelial–mesenchymal transition (EMT)-inducing transcription factor, is also upregulated in various malignant tumors. It plays a crucial role in EMT regulation by modulating the expression of CDH-1, OCLN, and CLDN-7, along with other effector genes, thereby facilitating cellular proliferation and differentiation (Hahn and Hermeking, 2014). *ZNF143* is a transcription factor implicated in the regulation of DNA and cell cycle-related genes. Pawel Sadlecki et al. investigated tissue samples from eight patients who were diagnosed with serous borderline tumors and 34 patients who were diagnosed with low-grade ovarian cancer. The findings demonstrated that the percentages of ZNF143- and ZNF281-positive were 90% and 57%, respectively (Sadlecki et al., 2019). Nevertheless, no statistically significant differences in the expression patterns of these transcription factors were observed between sBOTs and low-grade EOCs, which is possibly due to the fact that these two tumor types have a common origin and may have undergone dynamic EMT-related transformation processes (Sadlecki et al., 2019). *ZNF143* can directly transcriptionally activates the mineral dust-induced histone demethylase gene (MDIG), thereby enhancing the expression of cell division cycle 6 (CDC6) to facilitate the proliferation and colony formation of liver cancer cells (Zhang et al., 2020). Furthermore, the findings of Zhenyu Wang et al. suggested that the ZNF143-FBXO9-FBXW7 signaling axis may also serve as a pathway implicated in hepatocellular carcinoma development (Wang et al., 2022). According to Paek et al. (2010) the expression of ZNF143 in colorectal cancer cells is regulated by the IGF-1-PI3K signaling pathway (Figure 3C). However, the relevant molecular mechanisms in ovarian tumors remain to be further evaluated. Additionally, ZNF281 can interact with transcription factors such as NANOG, SOX2, and OCT4 to induce the expression of stem cell markers, including LGR5 and CD133*,* thereby facilitating the formation and pluripotency of tumor stem cells. Importantly, P53-mediated miRNAs exert a negative regulatory effect on *ZNF281* (Hahn and Hermeking, 2014) (Figure 3D). ZNF143 and ZNF281 may serve as novel targets for future cancer therapeutics.

#### 3.2.3 Cell damage repair factors

##### 3.2.3.1 CHEK2

**FIGURE 4:**
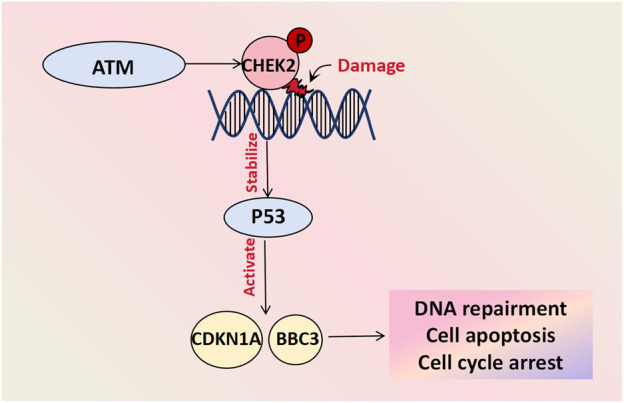
Cell damage repair molecules associated with BOT development. CHEK-2 undergoes rapid phosphorylation by upstream PI3K family members like the ATM gene in response to DNA damage and replication arrest. The activated CHEK2 protein stabilizes the inhibitory protein P53 and initiates downstream gene activation, including CDKN1A and BBC3, facilitating DNA repair, cell apoptosis and cycle arrest.

The CHE2 protein plays a crucial role in DNA damage repair, cell cycle regulation, and apoptosis. It acts as a cell cycle checkpoint regulator and tumor suppressor that undergoes rapid phosphorylation by upstream PI3K family members (such as the *ATM* gene) in response to DNA damage and replication arrest. This activation stabilizes the inhibition of the P53 protein and triggers downstream gene expression of CDKN1A and BBC3, among other factors, thus preventing damaged cells from entering mitosis and leading to G1 cell cycle arrest. Consequently, it induces DNA repair and cell apoptosis (Wang et al., 2018). Mutations in genes involved in the *ATM-CHEK2-P53* pathway have been shown to be closely associated with cancer risk (Kabacik et al., 2011). Genetic profiling analyses was conducted on 102 patients with BOTs and 1743 healthy controls and demonstrated that the presence of a missense mutation (c.470T>C) in the *CHEK2* gene increased the risk of developing BOTs by approximately two-fold. Furthermore, this mutation was associated with an earlier age at diagnosis and a decrease in the 10-year survival rate, thus suggesting its involvement in BOT development (Ogrodniczak et al., 2022) (Figure 4).

#### 3.2.4 Cell cycle regulators

##### 3.2.4.1 Retinoblastoma protein (Rb) family

**FIGURE 5:**
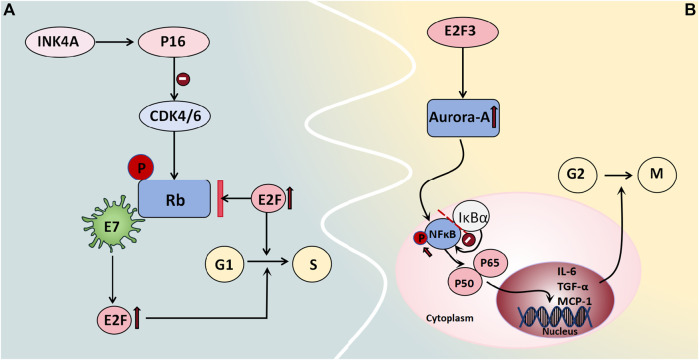
Cell cycle regulators associated with BOT development. **(A)** Rb binds to the transcription factor E2F as a growth inhibitor, regulating the cell cycle and tumor progression. Phosphorylation by CDKs causes Rb to dissociate from E2F, promoting E2F-mediated transcription and facilitating entry into the S phase of the cell cycle, thereby activating gene transcription and enhancing cell proliferation. The viral oncogene E7 also interacts with Rb protein to release E2F and facilitate cell cycle progression. **(B)** Aurora-A is regulated by E2F3 in transcription, mediating the carcinogenic function of E2F3. Aurora-A kinase phosphorylates NFκB, releasing it from its inhibitor IκBα and allowing translocation of the P65/P50 complex to the cell nucleus. This complex then acts on target genes like IL-6, TGF-α, and MCP-1 to regulate cell survival and apoptosis.

Rb/p105 is widely expressed in cells with cycling or quiescent nuclei. In its dephosphorylated state, it binds to the transcription factor E2F as a growth inhibitor, thus exerting control over the cell cycle and tumor progression and thereby playing a tumor-suppressive role (Amin et al., 2015). Phosphorylation of Rb/p105 by cyclin-dependent kinases (CDKs) disrupts its interaction with E2F, thus leading to enhanced E2F-mediated transcription and thereby facilitating cell entry into the S phase, which activates gene transcription and cell proliferation. The viral oncogene E7 also interacts with the Rb protein, thus releasing E2F and promoting cell cycle progression (Amin et al., 2015). Alterations in members of the Rb family are frequently associated with malignant gynecological tumors, whereas defects in the Rb pathway often correlate with poor clinical outcomes. Intracellular localization also regulates specific members of the Rb family. The expression levels and subcellular distribution patterns of proteins from the *Rb* gene family in 65 BOT patients were evaluated by Masciullo et al. (2020) and they demonstrated that approximately 61.6% of patients exhibited nuclear expression of Rb/p105, which was significantly greater among serous patients than among mucinous patients. Although benign tumors predominantly displayed nuclear localization of Rb2/p130, BOTs showed both nuclear and cytoplasmic expression patterns; moreover, malignant tumors primarily exhibited cytoplasmic expression. Low levels of nuclear expression coupled with high cytoplasmic levels were considered to be indicative of high-risk mucinous BOT progression and may serve as predictive factors for diagnosis and prognosis (Masciullo et al., 2020) (Figure 5A).

##### 3.2.4.2 Aurora-A kinase

Aurora-A kinase functions as a centrosome kinase and plays a crucial role in G2/M cell cycle progression, thus participating in the regulation of mitosis and the cell cycle. It also plays a significant role in centrosome and spindle maturation and assembly (He et al., 2008). Studies have demonstrated the gene amplification of Aurora-A in various tumors, including breast cancer, non-small cell lung cancer, colon cancer, and ovarian cancer. Differential expression levels of Aurora-A among normal Müllerian epithelium, benign and borderline serous/mucinous ovarian epithelial tumors, and malignant serous ovarian tumors were reported by Alkhateeb et al. (2021). The results showed that normal Müllerian epithelium and most benign tumors exhibited moderate-to-heavy nuclear staining without specific cytoplasmic staining. However, no nuclear immunoreactivity was observed in malignant serous ovarian tumors; moreover, only 42% of tissues displayed weak-to-moderate perinuclear cytoplasmic immunoreactivity. The characteristics of the borderline tumors were intermediate between those of these two groups. Two-way analysis comparing Aurora A immunoreactivity types (absent/cytoplasmic/nuclear) and intensity (strong/moderate/weak) for three tumor types demonstrated significant differences (except for borderline tumors versus malignant tumors) regarding staining intensity (Alkhateeb et al., 2021). This finding aligns with Heini Lassus et al.'s conclusion that solely cytoplasmic expression of Aurora-A is associated with tumor cell aneuploidy, whereas high Aurora-A expression strongly predicts short-term survival and poor patient prognosis (Lassus et al., 2011). Additionally, studies have demonstrated that the transcriptional regulation of Aurora-A is mediated by E2F3, thereby facilitating its carcinogenic function. Moreover, a positive correlation has been observed between the protein expression levels of Aurora-A and E2F3 in human ovarian cancer. The E2F3-Aurora-A axis may represent a crucial mechanism underlying ovarian tumor development and progression (He et al., 2008). Under normal conditions, NFκB is bound to its inhibitor IκBα and remains stable in the cytoplasm. When it is phosphorylated, it releases the P65/P50 complex and translocates to the nucleus, where it acts on target genes such as *IL-6, TGF-α*, and *MCP-1* to regulate cell survival and apoptosis. Chefetz et al. (2014) employed Aurora-A inhibitors to induce cell cycle arrest in ovarian tumor stem cells and demonstrated that these inhibitors enhance the nuclear accumulation of IκBα to suppress NFκB activity, thus suggesting a potential role for Aurora-A *k*inase in regulating cell cycle progression through the modulation of the NFκB pathway (Figure 5B).

#### 3.2.5 Tumor suppressor genes

Currently, extensive research has been conducted on protein patterns associated with cell proliferation and apoptosis in tumors, and the expression of tumor suppressor genes has been recognized to be closely linked to the occurrence, progression, and treatment response of neoplasms.

##### 3.2.5.1 P53 and related molecules

**FIGURE 6:**
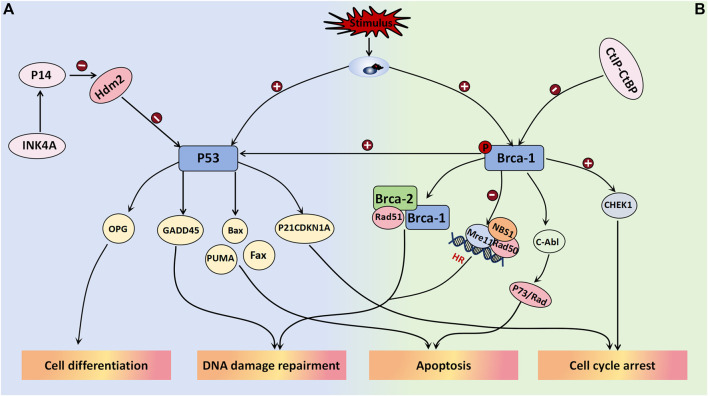
Tumor suppressor genes associated with BOT development. **(A)** P53 binds to specific DNA sequences of target genes in response to the cellular environment after activation, leading to various signal responses including cell cycle arrest (P21CDKN1A), apoptosis (Bax, Fax, PUMA), differentiation (OPG), and DNA damage repair (GADD45). It has been confirmed that p53 is part of the negative feedback loop involving HDM2 (MDM2 in mice). Inhibiting MDM2/MDMX effectively activates the p53 pathway in cancer cells overexpressing MDM2/MDMX. **(B)** BRCA1 forms a complex with BRCA2 and Rad51 to promote DSBS, while also regulating the Rad50-Mre11-NBS1 complex and inactivating Mre11 to play a crucial role in HR-mediated DSB. Additionally, it is involved in c-Abl activity regulation, leading to P73 or Rad-mediated cell apoptosis. Overexpression of BRCA-1 stimulates the P53 pathway, promoting the expression of response factors like P21 and GADD45 for its effects. In terms of cell cycle regulation, BRCA-1 activates Chk1 and its downstream signal transduction to regulate the G2/M checkpoint induced by DNA damage. BRCA-1 is weakened when bound by the CtIP-CtBP complex.

The tumor suppressor gene *P53* plays a crucial role in maintaining normal cellular growth and differentiation, and its association with the aggressiveness of ovarian tumors has been well established. Upon stimulation by DNA damage, hypoxia, and oncogene activation, among other stimuli, *P53* can be activated. Depending on the cellular environment, it binds to specific DNA sequences of various target genes to elicit distinct signaling responses, such as cell cycle arrest, apoptosis induction, and differentiation (Amin et al., 2015). In a study conducted by Cai et al. (2019), they utilized dimethylbenzanthracene to induce the development of a mouse model for mucinous borderline tumors. They observed a gradual increase in the expression level of P53 from mucinous benign ovarian tumors to mucinous borderline tumors and finally to mucinous ovarian cancer. These differences between groups were statistically significant, thus suggesting an intermediate pattern of P53 expression between benign and malignant ovarian tumors. Therefore, P53 may serve as a potential biomarker for grading mucinous ovarian tumors; however, further research is required to determine the specific threshold value. P53 has been demonstrated to participate in the negative feedback loop of HDM2 (MDM2 in mice), wherein MDM2 functions as an E3 ubiquitin ligase, thus leading to monoubiquitination and subsequent degradation of P53. This regulatory mechanism plays a pivotal role in controlling cell growth and has significant implications for the pathogenesis and metastasis of neoplasms. The absence of P14arf can result in an increase in the noncompetitive activity of MDM2, thereby promoting tumor cell proliferation and conferring enhanced resistance to immunotherapy. The targeting of the MDM2/MDMX axis represents a promising strategy to reactivate the P53 pathway in cancer cells overexpressing MDM2/MDMX, thus offering a novel avenue for current cancer therapeutics (Corney et al., 2008). The activation of the transcription factor P21WAF1/CIP1, which is a cell cycle protein-dependent kinase, may represent one of the mechanisms by which P53 regulates the cell cycle. As a downstream molecule in the P53 signaling pathway, it can impede the transition from the G1 phase to the S phase, thus resulting in cell cycle arrest (Amin et al., 2015; Palazzo et al., 2000). In a study conducted by JUAN R. PALAZZO et al., involving 40 patients with BOTs, 80% of these tumors exhibited positive expression of P21WAF1/CIP1, which is significantly greater than that of both ovarian cancer and benign ovarian tumors. Additionally, 90% of these tumors showed positive MDM2 expression; however, no significant differences were noted in MDM2 expression across different tumor types. Interestingly, there was a remarkably high coexpression rate (80.6%) between P21WAF1/CIP1 and MDM2 in serous borderline ovarian tumors compared to both benign and malignant ovarian tumors, as well as mucinous borderline tumors (Palazzo et al., 2000). Notably, the Rb pathway appears to interact with the P53 pathway, which is likely due to the INK4a family of cyclin-dependent kinase inhibitors encoding two proteins known as P16 and P14. The former protein inhibits CDK4/6 activity, thereby affecting Rb phosphorylation, whereas the latter protein enhances P53 expression by inhibiting HDM2. The study demonstrated that overexpression of E2F in P14-deficient cell lines induced S phase entry, whereas low levels of E2F upregulated P14 expression to regulate cell cycle progression (Corney et al., 2008) (Figure 6A).

##### 3.2.5.2 BRCA-1

The *BRCA-1* gene, which is located on chromosome 17, is a crucial tumor suppressor involved in DNA mismatch repair and double-strand break repair processes and plays a pivotal role in maintaining genomic stability. Extensive research has focused on *BRCA-1* gene mutations across various malignancies, such as breast cancer, lung cancer, and ovarian cancer. Its regulatory function is believed to be associated with the modulation of the Wnt/β-catenin and PI3K/PTEN/AKT signaling pathways. Immunohistochemical staining on 214 ovarian tumor patients was performed to investigate the findings, thus demonstrating a gradual decrease or even disappearance of *BRCA-1* gene expression in benign-borderline-malignant ovarian tumors (Yousif and Mohammed, 2019). However, further investigation is required to determine the expression threshold between each category. The downregulation of *BRCA-1* gene expression in serous tumors indicates a higher tumor histological grade, degree of necrosis, and proliferation index (Yousif and Mohammed, 2019). When DNA is damaged, BRCA-1 can be phosphorylated at multiple sites by various kinases, thereby participating in the activation of double-strand break repair and initiation of homologous recombination. BRCA-1 forms a complex with BRCA-2 and Rad51 to facilitate DSBS promotion while also acting as a regulator of the Rad50-Mre11-NBS1 complex, thus playing a crucial role in HR-mediated DSBs through Mre11 inactivation. Moreover, it is believed that BRCA-1 is involved in regulating c-Abl activity, thus consequently inducing P73- or Rad-mediated cell apoptosis (Yoshida and Miki, 2004). The overexpression of BRCA-1 can stimulate the P53 pathway to enhance the expression of response factors such as P21 and GADD45, thus exerting its effects. In the regulation of cell cycle progression, BRCA-1 activates Chk1 and its downstream signal transduction pathway to govern the G2/M checkpoint response triggered by DNA damage. Additionally, research has indicated that the CtIP-CtBP complex regulates BRCA-1, and their binding often attenuates BRCA-1 activation (Yoshida and Miki, 2004). Notably, olaparib (which is a PARP inhibitor) represents a targeted therapeutic drug that is currently employed in patients with ovarian tumors harboring *BRCA-1* gene mutations, thus yielding favorable clinical outcomes (Figure 6B).

## 4 Treatment

Currently, the main treatment method for BOTs involves surgery. According to relevant statistics, the incidence rate of borderline ovarian tumors accounts for 10%–20% of epithelial ovarian tumors, and half of the patients have not completed childbearing. For this group of people, the preservation of fertility surgery can not only give them the opportunity to have children but also significantly improve the quality of life and the happiness index of patients after surgery. However, most studies have indicated that the risk of recurrence is significantly greater for BOT patients after fertility-preserving surgery. Sozen H. conducted a retrospective evaluation of 103 BOT patients, and the results showed that fertility-preserving surgery is a significant independent predictor of recurrence and progression of BOTs, with a 94-fold increase in the risk of recurrence; however, no significant correlation was observed between lymph node dissection and the recurrence rate (Sozen et al., 2018). A meta-analysis conducted by Li et al. (2020) demonstrated that patients who underwent unilateral cystectomy for BOT had a significantly greater risk of recurrence than those who underwent unilateral salpingo-oophorectomy, whereas no significant difference was observed in pregnancy outcomes. Despite the high recurrence rate following conservative surgery for BOT patients, the majority of recurrences are borderline and confined to the ovary, thus exerting minimal impacts on OS. Nevertheless, these patients should receive more intensive long-term follow-up after surgery.

In terms of the surgical approach, the conventional belief is that laparoscopic surgery is associated with a greater risk of tumor rupture, incision site metastasis, and unclear staging than open surgery. However, with recent advancements in laparoscopic technology, especially in early BOT conservation surgery, laparoscopic surgery is now considered to offer equivalent safety and therapeutic benefits to traditional open surgery. Furthermore, it results in reduced intraoperative bleeding and postoperative complications while facilitating faster patient recovery (Bourdel et al., 2021; Li et al., 2020). Currently, numerous scholars believe that chemotherapeutic drugs exhibit high sensitivity toward proliferative active cells (malignant tumor cells) but low sensitivity toward low proliferative BOTs; moreover, they may even demonstrate anti-chemotherapeutic effects. Consequently, adjuvant radiotherapy and chemotherapy are not currently recommended for BOT patients.

## 5 Conclusion

BOTs are classified as low-grade, potentially malignant neoplasms, and they occupy an intermediate position between benign and malignant tumors, thereby posing significant clinical challenges in terms of accurate diagnosis. With the increasing understanding of molecular markers and mechanisms associated with BOTs, precision diagnosis for patients has improved. This study aimed to comprehensively investigate and summarize the recent advancements in molecular biology and associated mechanisms pertaining to BOTs, thereby providing some help and guidance for the differential diagnosis and treatments. For BOTs, further research is required to fully comprehend the current molecular mechanisms that are incompletely understood at present and to identify more sensitive and specific diagnostic markers in the future.
